# Divergent Microbial Community and Pathogenicity at a University-Urban Interface: A Comparative Analysis

**DOI:** 10.3390/microorganisms14030557

**Published:** 2026-02-28

**Authors:** Xinyu Liu, Nan Xiao, Jianghao Yu, Xueyun Geng, Mengge Zhang, Youming Zhang, Hai Xu, Changliang Nie, Mingyu Wang, Ling Li

**Affiliations:** 1 State Key Laboratory of Microbial Technology, Microbial Technology Institute, Shandong University, Qingdao 266237, China; 2 Shanghai Key Laboratory of Atmospheric Particle Pollution and Prevention (LAP), Shanghai 200438, China; 3 School of Environment and Geography, Qingdao University, Qingdao 266071, China

**Keywords:** university–urban interface, environmental metagenomics, antimicrobial resistance, hemolytic activity, methicillin-resistant staphylococci

## Abstract

Environmental metagenomics and microbial taxonomy provide essential frameworks to evaluate how population structures shape the evolution of antimicrobial resistance and microbial community dynamics within densely populated environments. To evaluate microbial community composition and pathogenic potential, high-touch surfaces at high-traffic sites on and off campus were analyzed using metagenomics and characterization of 188 bacterial isolates, including antibiotic susceptibility testing, hemolytic assays, and whole-genome sequencing. Off-campus sites showed significantly higher bacterial richness and more complex communities enriched with diverse potential pathogens. Notably, high-risk carbapenemase genes were predominantly identified in these off-campus urban environments. In contrast, on-campus environments harbored less diverse communities dominated by opportunistic, antibiotic-resistant *Staphylococcus* species, with metagenomic analysis confirming a concentrated enrichment of β-lactam resistance determinants associated with methicillin-resistant staphylococci. Phenotypic profiling revealed extensive antimicrobial resistance, with 84.7% of isolates exhibiting resistance to at least one antibiotic and 35.1% of *Staphylococcus* showing hemolytic activity. Whole-genome sequencing further revealed that these resistance and pathogenic traits are predominantly localized on mobile plasmids, highlighting a high potential for horizontal gene transfer. These findings indicate that population activities shape distinct microbial communities in closely adjacent environments and highlight the importance of monitoring high-risk resistance determinants in densely populated university settings.

## 1. Introduction

Antimicrobial resistance (AMR) has emerged as a critical global health challenge, with bacterial infections causing over 1.27 million deaths in 2019 [1] and projected to result in 10 million annually by 2050 [2]. AMR has been widely detected across diverse environments, ranging from clinical infections [3] and hospital wastewater [4] to aquaculture farms [5] and campus air environments [6], demonstrating that resistance is ubiquitous across human activity scenarios and requires urgent attention.

Most current AMR research has focused on clinical or public environments, whereas campuses and their surrounding areas remain understudied despite their unique features—dense, mobile populations engaged in frequent group activities. However, several studies [7,8] have described bacterial outbreak events in educational settings, such as infectious gastroenteritis caused by *Escherichia coli* infection and skin infections caused by *Staphylococcus*. Such occurrences, coupled with evidence that frequently touched surfaces on campus serve as significant environmental reservoirs for resistant bacteria [9], highlight the potential for human-mediated transmission within these high-density settings. Furthermore, human-impacted environments have been shown to act as reservoirs that promote the enrichment and horizontal transfer of antibiotic resistance genes (ARGs), significantly shaping the environmental resistome [10]. This underscores the critical role of human-mediated dissemination in shaping the environmental resistome, particularly in high-density areas such as university campuses. Following the global COVID-19 pandemic, population mobility has largely resumed; however, many Chinese universities have maintained specific visitor entry application requirements based on institutional management directives [11], resulting in distinct demographic structures that may further influence microbial community dynamics. To examine how these demographic shifts, alongside holiday-related movements, shape microbial communities and AMR distribution, we selected a university in Qingdao as the study site. The campus hosts a stable population of approximately 14,000 residents [12], while the adjacent community—home to roughly 60,000 inhabitants—is situated near a seaside park that attracts high volumes of tourists [13]. This leads to more complex and transient off-campus populations compared to the relatively homogeneous on-campus group. Therefore, studying changes in microbial communities and resistance distribution in these distinct environments is of great significance for monitoring the environmental reservoir of opportunistic pathogens.

With advances in sequencing technologies, resistome characterization has become increasingly feasible [14,15], revealing the widespread presence of ARGs across human-impacted environments. In this study, we integrated metagenomic sequencing with antimicrobial resistance and hemolysis testing of culturable bacteria to compare microbial communities on frequently touched surfaces in on- and off-campus environments, aiming to evaluate bacterial risks and their potential role in antimicrobial resistance spread. This study provides new insights into how differences in population dynamics between on- and off-campus shape microbial communities and resistance risks, with numerous resistant and hemolytic pathogenic bacteria detected from isolated strains, highlighting the need for continuous monitoring of high-contact surfaces in densely populated settings.

## 2. Materials and Methods

### 2.1. Sample Collection

Samples were collected from the campus and surrounding environment of Shandong University in Qingdao, Shandong Province. Thirteen sampling locations were selected, including areas with high foot traffic such as the express delivery center, commercial center, restaurants, subway station, etc. Swabs were taken from frequently touched surfaces, including door handles, toilets, and elevators, etc. Swabs were pre-moistened with sterile saline and used to swab sampling surfaces with multiple passes. The swabs were then placed into 2 mL centrifuge tubes and temporarily stored in ice boxes. Sampling was conducted once before and once after the International Labor Day holiday, with an interval of four days, collecting a total of 58 samples. After adding 1 mL of saline to each tube, the samples were vortexed for 1 min to create bacterial suspensions for subsequent bacterial isolation.

### 2.2. Whole Genomic DNA Extraction

Centrifuge tubes containing bacterial suspensions and swabs were centrifuged to remove the supernatant. Then, 1 mL of CTAB lysis buffer (Tris (100 mM), NaCl (1.4 M), EDTA (20 mM), CTAB (2%), pH = 8.0) and lysozyme were added, followed by incubation at 65 °C in a water bath. The supernatant was transferred to a new tube and extracted sequentially with phenol (pH 8.0):chloroform:isoamyl alcohol (25:24:1) and chloroform:isoamyl alcohol (24:1). DNA was precipitated with an equal volume of isopropanol at −20 °C, pelleted by centrifugation, washed with 70% ethanol, airdried, and dissolved in ultrapure water. Extracted DNA was stored at −20 °C until further use.

### 2.3. Metagenomic Sequencing, Quality Control, and Assembly

Each sample used 0.2 μg of DNA for DNA library preparation. The genomic DNA was fragmented to 350 bp in size by sonication. The DNA fragments underwent end-polishing, A-tailing, and ligation with full-length adapters for sequencing, followed by PCR amplification. The PCR products were purified using the AMPure XP system (Beckman Coulter, Beverly, MA, USA). Library quality was assessed using the Agilent 5400 system (Agilent, Santa Clara, CA, USA) and quantified by qPCR (1.5 nM). Different libraries were pooled based on their effective concentrations and the required data amount. The 5′ end of each library was phosphorylated and cyclized, followed by loop amplification to generate DNA nanoballs. These DNA nanoballs were loaded onto the MGI DNBSEQ-T7 platform for sequencing. Each sample generated data according to the 10 Gb standard, with specific sample data quantities listed in Table S1. Quality control of raw reads was performed using fastp (v0.20.0) [16], to remove adapters and low-quality reads. Subsequently, Bowtie2 (v2.3.5.1) [17] to remove host contamination by aligning reads against the human reference genome (hG38/GRCh38) with default parameters. Individual metagenomic assembly was performed for each sample using a custom batch script with Megahit (v1.1.3) [18].

### 2.4. Bioinformatics Analysis Process

Species annotation of the assembled sequences was performed using Kraken2 (v2.0.9) [19]. After obtaining the microbial distribution table, Alpha-diversity and β-diversity analyses were performed. Principal Coordinate Analysis (PCoA) and Non-metric multidimensional scaling (NMDS) were used for dimensionality reduction of microbial community data. LDA Effect Size (LEfSe, v1.0.8) [20] was applied to observe microbial enrichment across different sampling locations. PlasFlow (v1.1.0) [21] was used to predict plasmid sequences from the assembled final fragments. AMRFinderPlus (v4.0.23) [22] was used to predict ARGs in the assembled final fragments and plasmids. To ensure comparability across samples, the abundance of each identified ARG was quantified using FPKM (Fragments Per Kilobase of transcript per Million mapped reads), which normalizes for differences in gene length and sequencing depth. The FPKM for each ARG was calculated as: FPKM = (R × 10^9^)/(L × T), where R is the read count (estimated as average coverage × gene length), L is the gene length in base pairs, and T is the total number of mapped reads for all genes. For category-level analysis, FPKM values of all individual genes within each respective category were summed. The relationship between resistance genes and plasmids was analyzed to evaluate the distribution, infection risk, and transfer potential of antibiotic resistance genes.

### 2.5. Bacterial Isolation and Antimicrobial Resistance/Hemolysis Assays

Aliquots (100 μL) of bacterial suspension obtained by vortexing the samples were streaked on LB agar plates and incubated at 37 °C for 16 h. Bacteria with different colony morphologies were transferred to LB liquid medium and cultured overnight with shaking, and glycerol was added for bacterial preservation. Bacterial identification was performed using the 27F/1492R primer pair. Antibiotic sensitivity testing was performed with eight antibiotics with different mechanisms of action, following CLSI and EUCAST standards. Specifically, cefoxitin resistance was utilized as a phenotypic surrogate to determine methicillin resistance in *Staphylococcus* isolates. Quality control strains used in antimicrobial susceptibility testing included *Escherichia coli* ATCC 25922, *Staphylococcus aureus* ATCC 25923, and *Enterococcus faecalis* ATCC 29212. *Bacillus* and *Staphylococcus* species were selected to test their hemolytic activity. After overnight bacterial culture, the bacterial suspensions were diluted 1000 times in sterile PBS and inoculated onto blood agar plates (Haibo Biotechnology Co., Qingdao, China).

### 2.6. Single-Strain Genome Sequencing and Analysis

Single bacterial strains were cultured overnight, and genomic DNA was extracted as described above. Sequencing libraries were prepared using the Rapid Barcoding Kit 96V14 (SQK-RBK114.96, Oxford Nanopore Technologies, Oxford, UK) following the manufacturer’s instructions. Sequencing was performed on a Nanopore P2 Solo system with an R10.4.1 flow cell. Genome assembly was performed with Flye (v2.8.1-b1676) [23] and sequence polishing with Medaka (v1.12.0). Assembly completeness was evaluated using BUSCO (v5.2.2) [24]. The remaining analysis steps and software used were the same as described above.

### 2.7. Statistical Analysis

Alpha-diversity indices were compared using Student’s *t*-test for normally distributed data and the Mann–Whitney *U* test for non-normally distributed data. β-diversity was assessed with PCoA and NMDS based on Bray–Curtis dissimilarity, with group differences tested by PERMANOVA. Differentially abundant taxa were identified by LEfSe using Kruskal–Wallis test with Wilcoxon rank-sum pairwise comparisons (LDA > 2.0, *p* < 0.05). Antibiotic resistance gene abundances were compared using Student’s *t*-test, Welch’s *t*-test, or the Mann–Whitney *U* test, depending on data normality and variance homogeneity. Relative abundance data were standardized using Z-score transformation where appropriate. Ecological distance comparisons were conducted with Wilcoxon rank-sum tests, and statistical significance was set at *p* < 0.05.

## 3. Results

### 3.1. Microbial Community Structure Analysis

**Figure 1:**
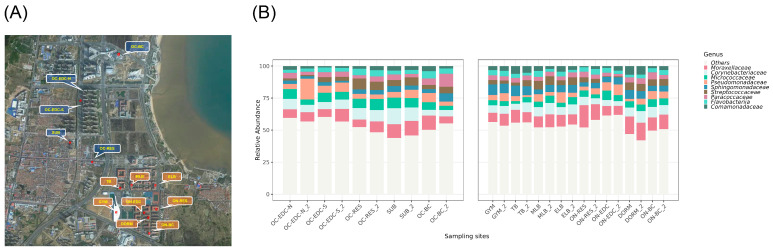
Sampling site location map and bacterial community structure. (**A**) Distribution map of sampling site locations. Sampling sites in the figure are divided into off-campus and on-campus groups. The abbreviations and brief descriptions for each site are as follows: Blue labels represent off-campus sampling group, including OC-EDC-N: Off-campus express delivery center-North; OC-EDC-S: Off-campus express delivery center-South; OC-RES: Off-campus restaurants; SUB: Subway station; OC-BC: Off-campus Business Center. Orange labels represent on-campus sampling group, including GYM: Gymnasium; TB: Teaching Building; MLB: Microbiological Laboratory Building; ELB: Engineering Laboratory Building; ON-RES: On-campus restaurants; ON-EDC: On-campus express delivery center; DORM: Student dormitory; ON-BC: On-campus Business Center. (**B**) Genus-level bacterial community structure at each sampling site. The figure shows the top nine most abundant genera in bacterial communities at each location.

Thirteen sampling sites were selected within a ~1.5 km radius, including eight on-campus and five off-campus locations with high human traffic (Figure 1A). Several sites had comparable functions across campus boundaries, such as express centers, restaurants, and commercial centers, enabling direct comparisons.

Metagenomic sequencing was used to assess bacterial community composition. At the phylum level, Pseudomonadota dominated all samples (38.8–58.8%), followed by Actinomycetota (20.0–43.3%) and Bacillota (7.13–18.9%), while other phyla were less abundant. At the genus level (Figure 1B), *Corynebacterium*, *Pseudomonas*, and *Acinetobacter* were most abundant, all of which include important pathogens such as *Corynebacterium diphtheriae*, *Pseudomonas aeruginosa*, and *Acinetobacter baumannii*. Other abundant genera, including *Streptococcus*, *Staphylococcus*, and *Moraxella*, also harbor pathogenic species. Since samples were collected from frequently touched surfaces in densely populated areas, these findings suggest potential risks of bacterial transmission and infection, warranting further evaluation of pathogenic species.

### 3.2. Significant Differences in Microbial Communities Between On- and Off-Campus

**Figure 2:**
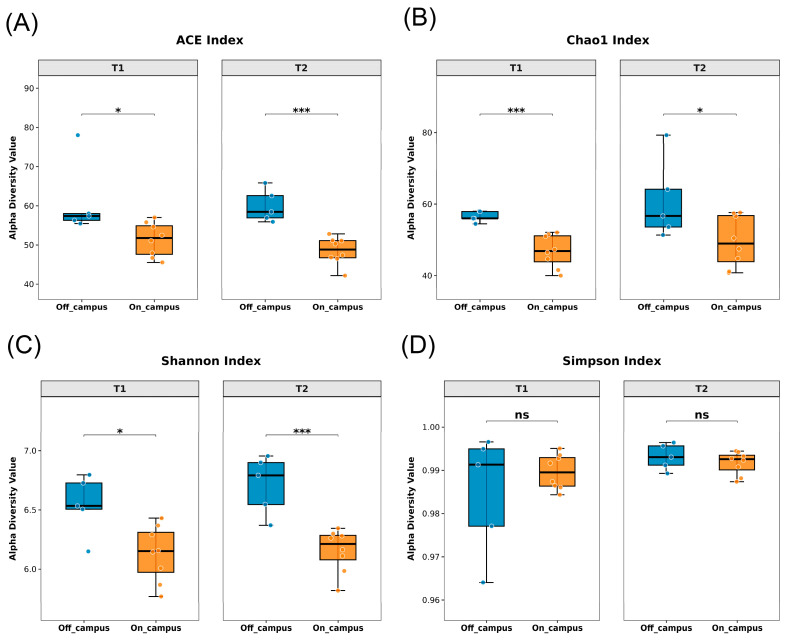
Microbial community Alpha- diversity analysis. (**A**) ACE index of microbial communities; (**B**) Chao1 index of microbial communities; (**C**) Shannon index of microbial communities; (**D**) Simpson index of microbial communities. Each sampling site was grouped into on-campus and off-campus categories, with coloring according to grouping: off-campus in blue and on-campus in orange. Comparisons were made at two sampling times to observe differences in Alpha-diversity between on- and off-campus. Statistical significance was determined using Student’s *t*-test for normally distributed data (Chao1, Shannon, Simpson) and Mann–Whitney *U* test for non-normally distributed data (ACE). Significance markers are: ns, not significant; *, *p* < 0.05; ***, *p* < 0.001.

Alpha-diversity indices (ACE, Chao1, Shannon, Simpson) were calculated for each site (Figure 2). Off-campus locations showed significantly higher ACE, Chao1, and Shannon values compared to on-campus sites. Notably, although no significant differences were observed in the Simpson index between the two environments, the higher values of the other three indices consistently highlight the distinct microbial profiles off-campus. No significant differences were observed between the two sampling times, suggesting short-term stability of community structures. Higher diversity may be associated with sites experiencing heavier human traffic, such as off-campus Express Delivery Center (EDC) and subway stations, as well as on-campus EDCs, commercial centers, and restaurants. Overall, off-campus environments exhibited higher bacterial richness, reflecting differences in human activity levels.

**Figure 3:**
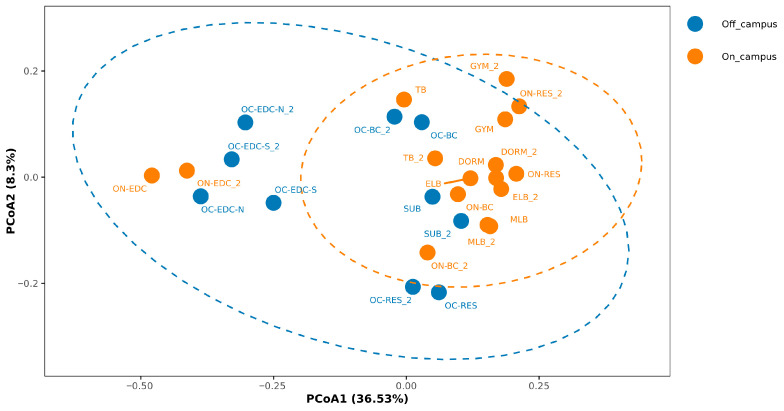
PCoA analysis of microbial communities in on- and off-campus environments. PCoA based on Bray–Curtis distance, where each point represents a sample, colored according to grouping: off-campus in blue and on-campus in orange. Inter-group differences were statistically tested using PERMANOVA (999 permutations), *p* = 0.020, showing statistical significance.

β-diversity analysis using PCoA and NMDS revealed significant compositional differences between on- and off-campus environments (PCoA, *p* = 0.020; Figure 3 and Figure S1). Samples from the same site clustered together, indicating short-term stability of community structures, which was consistent with Alpha-diversity results (Figure S1). Notably, a distinct cluster was formed by samples from EDC, comprising representatives from both on- and off-campus environments. Shared logistics systems and uniform handling procedures likely homogenize the microbial profiles of EDCs, leading them to cluster together regardless of their on- or off-campus locations. Collectively, off-campus sites exhibited higher bacterial richness and distinct microbial community structures compared with on-campus sites, which was related to the characteristics of the active population.

### 3.3. Microbial Enrichment and Pathogenicity Analysis

**Figure 4:**
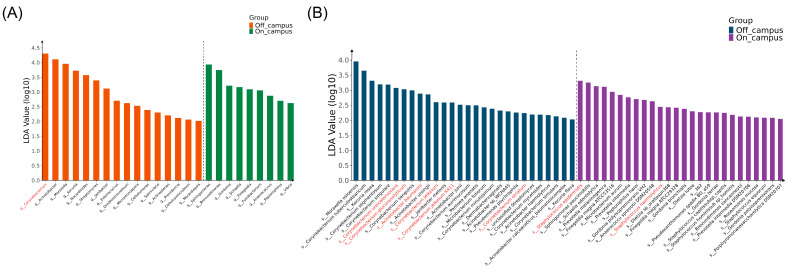
LEfSe analysis reveals distinct pathogenic microbial enrichment patterns on- and off-campus. (**A**) Genus-level high-risk taxa. Significantly enriched genera with elevated pathogenic potential between on-campus and off-campus environments. (**B**) Species-level high-risk taxa. Significantly enriched species with elevated pathogenic potential between on-campus and off-campus environments. All analyses show significant differential enrichment (LDA > 2.0, *p* < 0.05) determined using Kruskal–Wallis test with Wilcoxon rank-sum pairwise comparisons. Taxa highlighted in red represent clinically relevant pathogens discussed in the article. For all panels, the x-axis represents taxonomic information and the y-axis represents LDA scores.

LEfSe analysis identified significant differences in microbial enrichment between on- and off-campus groups, particularly at the genus and species levels. Species with preliminary assessment of pathogenic potential are shown in Figure 4, while others are presented in Figure S2. Off-campus sites were enriched with more taxa (157 vs. 73), including clinically important pathogens such as *Corynebacterium jeikeium*, *Corynebacterium striatum* [25,26], *A.baumannii*, and *Acinetobacter pittii* [27,28], indicating broad pathogenic risks. On-campus sites showed enrichment of fewer taxa overall but had a higher proportion of pathogenic microorganisms, dominated by opportunistic pathogens such as *Staphylococcus haemolyticus* and *Staphylococcus epidermidis* [29]. These findings suggest that while off-campus environments harbor greater diversity and a wider range of pathogens due to more complex human activities, on-campus environments remain high-risk because of the dominance of drug-resistant staphylococci.

In summary, despite notable differences in microbial enrichment patterns between campus and surrounding areas, potentially harmful species were detected in both environments, underscoring the need for further assessment of microbial pathogenic risks in these densely populated settings.

### 3.4. Widespread Distribution of Antibiotic Resistance Genes

**Figure 5:**
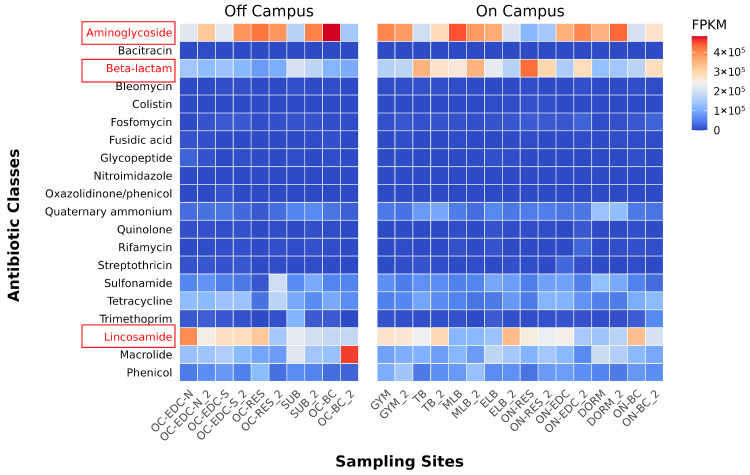
Abundance heatmap of antibiotic resistance gene categories. The heatmap illustrates the abundance profiles of various ARG categories across sampling sites, faceted into off-campus and on-campus groups. The horizontal axis represents antibiotic resistance categories, and the vertical axis represents the sampling sites. Categories highlighted in red (red text and boxes) indicate those associated with methicillin resistance and the broader multidrug-resistant profiles typical of methicillin-resistant staphylococci.

AMRFinderPlus prediction identified ARGs against 21 major antibiotic classes in all samples, with each site harboring over 10 distinct types (Figure 5, Table S2). Aminoglycoside resistance genes were most abundant, while Bacitracin resistance was rare. Sites with heavy human traffic, particularly off-campus locations like subway stations, express centers, and commercial centers, exhibited significantly higher ARGs abundance. Multiple last-resort ARGs were detected (Table S2), including carbapenemases (notably *bla*_KPC_, *bla*_GES_, and several *bla*_IMP_ variants), polymyxin resistance genes (*mcr-3*, *mcr-5*, *mcr-10*), glycopeptide resistance (*vanR*, *vanC1*), and linezolid resistance, though no resistance was found for tigecycline or daptomycin. Crucially, several high-risk carbapenemase determinants, such as *bla*_IMP-1_, *bla*_OXA-246_, and *bla*_OXA-275_, were identified as plasmid-borne (Table S2), suggesting a high potential for horizontal gene transfer (HGT) via frequently touched surfaces and human contact. The presence of these clinically significant genes in diverse urban environments underscores a potential public health risk that extends beyond common commensal bacteria.

**Figure 6:**
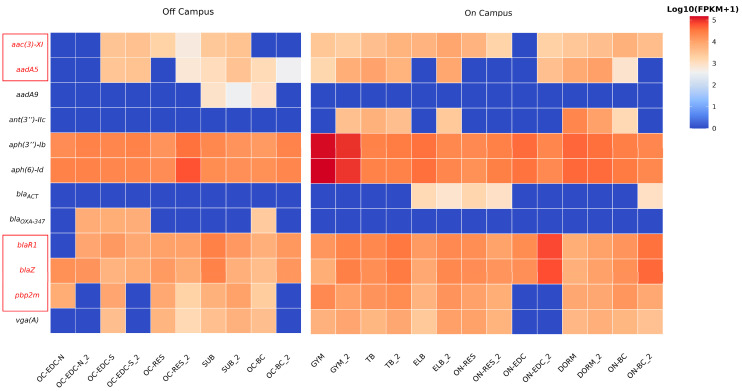
Abundance heatmap of three categories of antibiotic resistance genes. Resistance genes from aminoglycoside, β-lactam, and lincosamide antibiotic categories were selected for statistical analysis. Statistical significance was determined using Student’s *t*-test, Welch’s *t*-test, or the Mann–Whitney *U* test, depending on data normality and variance homogeneity. Results are displayed grouped by on- and off-campus, showing 12 resistance genes. To avoid excessive abundance differences between different genes, values are displayed as log(FPKM+1) transformed FPKM values. Gene names highlighted in red (with red text and red boxes) indicate genes that may contribute to methicillin resistance.

Heatmap and statistical analyses revealed a significantly higher abundance of β-lactam resistance genes on-campus at both sampling times. Within this category, we specifically identified determinants associated with methicillin and penicillin resistance at multiple on-campus locations. Combined with LEfSe analysis showing abundant *Staphylococcus* enrichment on-campus, we investigated the possible Methicillin-Resistant staphylococci (MRS) emergence at the resistance gene level. Statistical testing of genes within aminoglycoside, β-lactam, and lincosamide resistance categories (Figure 6) revealed that MRS-associated ARGs, including determinants for β-lactam resistance (*blaR1*, *blaZ*, *pbp2m*) and genes contributing to multi-drug resistance (*aac(3)-XI*, *aadA5*, *vga(a)*), were predominantly found on-campus, indicating a need to monitor MRS-related risks in these environments. Overall, ARGs against nearly all antibiotic classes were detected across sites, with last-resort resistance genes widely distributed and frequently plasmid-borne, suggesting strong potential for HGT.

### 3.5. Antibiotic Resistance and Hemolysis of Cultivable Bacteria

**Figure 7:**
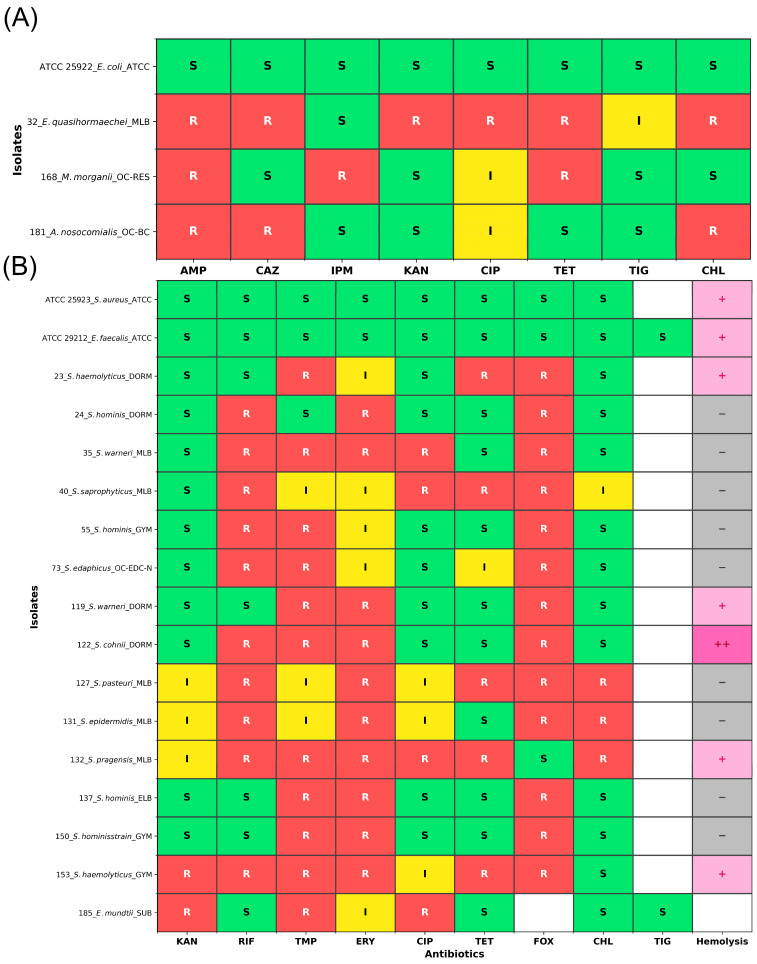
Antimicrobial susceptibility profiles and hemolytic activity of multidrug-resistant bacterial isolates. (**A**) Antimicrobial susceptibility heatmap of Gram-negative bacteria isolates. Only isolates resistant to ≥3 classes of antimicrobial agents were included. Antimicrobial agents tested: AMP (ampicillin), CAZ (ceftazidime), IPM (imipenem), KAN (kanamycin), CIP (ciprofloxacin), TET (tetracycline), TIG (tigecycline), CHL (chloramphenicol). Color coding: bright green indicates susceptible (S), bright yellow indicates intermediate (I), bright red indicates resistant (R), and white indicates no data. (**B**) Antimicrobial susceptibility heatmap and hemolytic activity profiles of Gram-positive bacteria isolates. Only isolates resistant to ≥3 classes of antimicrobial agents were included. Antimicrobial agents tested: KAN (kanamycin), RIF (rifampin), TMP (trimethoprim), ERY (erythromycin), CIP (ciprofloxacin), TET (tetracycline), FOX (cefoxitin), CHL (chloramphenicol), TIG (tigecycline). For antimicrobial susceptibility, color coding is the same as panel A. For hemolytic activity: gray background with “−” indicates no hemolytic activity, light pink background with “+” indicates hemolytic activity, dark pink background with “++” indicates strong hemolytic activity, and white background indicates not tested. Isolate labels are formatted as: Strain number_Species name_Sampling site.

A total of 188 culturable bacterial strains were isolated, including 85 clinically relevant strains (e.g., *Enterobacter*, *Acinetobacter*, and *Staphylococcus*) that underwent antimicrobial susceptibility testing (Table S3). Among them, 72 strains (84.7%) were resistant, with higher resistance rates on-campus (87.0%) than off-campus (75.0%). Eighteen strains (21.2%) were multidrug-resistant (MDR). Figure 7 displays the antimicrobial resistance and hemolytic patterns of these MDR isolates. Notably, two Gram-negative strains were carbapenem-resistant, and Gram-positive strains showed high resistance to rifampicin (21.3%), trimethoprim (45.9%), and erythromycin (55.7%). Hemolysis testing revealed that 35.1% of *Staphylococcus* and *Bacillus* strains were hemolytic, with some exhibiting strong hemolytic activity (Figure S3), indicating their pathogenic potential.

Whole-genome sequencing of selected MDR or hemolytic strains showed consistency between phenotypes and genotypes (Table S2), with frequent detection of *msr(A)*, *oqxB*, and *tet(K)* genes. β-lactam resistance genes were most abundant, suggesting significant risks to this clinically important antibiotic class. Eleven of 13 sequenced strains harbored plasmids, and nearly half carried plasmid-borne resistance genes, often linked to mobile genetic elements, indicating high potential for transmission. Among the seven sequenced *Staphylococcus* strains, six (85.7%) harbored plasmids carrying resistance genes, including β-lactam resistance genes (*blaZ*, *blaR1*, *blaI*), tetracycline resistance genes (*tet(K)*), and macrolide resistance genes (*mph(C)*, *msr(A)*) (Table S2). Notably, multiple strains (e.g., strains 35, 40, 127, 132) carried identical resistance genes on both chromosomes and plasmids, suggesting active HGT. The abundance of plasmid-mediated β-lactam resistance determinants may facilitate the emergence and spread of MRS in these environments.

In summary, resistant and pathogenic strains were widespread, with higher overall resistance rates on-campus but higher MDR prevalence off-campus, suggesting distinct selection pressures in different environments. The prevalence of plasmid-mediated ARGs and evidence of HGT further underscore the risk of rapid dissemination in this region.

### 3.6. Potential Prevalence of MRS in On-Campus Environments

**Figure 8:**
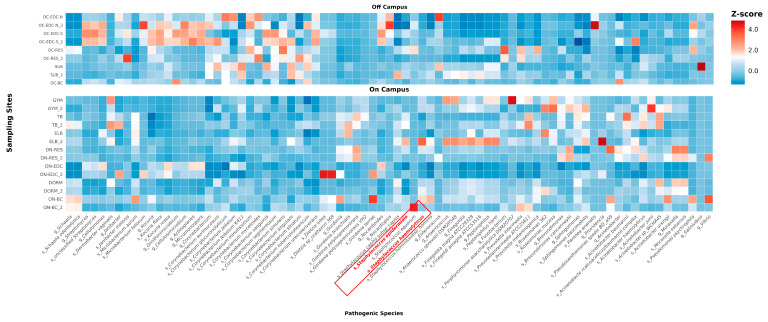
Genus- and species-level pathogen distribution heatmap. Species identified as pathogens in LEfSe analysis were extracted and displayed in two facet plots for on- and off-campus groups, with horizontal axis showing species information and vertical axis showing sampling locations. To facilitate observation of species enrichment patterns and eliminate differences in absolute abundance values, species abundance at each sampling site was standardized using Z-score transformation. Positive Z-scores (red) indicate relatively high abundance, while negative Z-scores (blue) indicate relatively low abundance. Species highlighted in red (with red text and red boxes) represent opportunistic *Staphylococcus* species.

Both metagenomic analysis and culture-based isolation consistently revealed significant enrichment of *Staphylococcus* in on-campus environments. LEfSe analysis identified two opportunistic *Staphylococcus* species, *S. epidermidis* and *S. haemolyticus*, as significantly enriched at multiple on-campus sites (Figure 8), and a large number of cultured isolates of these species were also detected on-campus (Table S3). The proportion of resistant *Staphylococcus* was higher on-campus (93.2%) compared to off-campus (77.8%), with on-campus strains exhibiting a higher hemolytic rate (34.1% vs. 11.1%), suggesting enhanced virulence potential.

Multiple cefoxitin-resistant strains, a phenotypic marker of MRS, were isolated from on-campus sites. Metagenomic analysis further detected abundant determinants associated with multidrug-resistant profiles of MRS, including resistance genes against β-lactams, aminoglycosides, and lincosamides (Figure 6, Table S2). As noted above, whole-genome sequencing revealed that the majority (85.7%) of *Staphylococcus* isolates harbored plasmids carrying penicillinase operon (*blaZ*, *blaR1*, *blaI*), with several strains showing evidence of recent HGT through chromosomal-plasmid gene duplication. This high prevalence of mobile resistance determinants poses substantial risks for rapid HGT and MRS dissemination.

In conclusion, the significant enrichment of resistant *Staphylococcus* species, including potential MRS strains, in on-campus environments highlights the substantial risk of transmission and infection, particularly given the documented plasmid-mediated dissemination mechanisms in these densely populated settings. These findings emphasize the need for enhanced surveillance and infection control measures on campus.

## 4. Discussion

This study investigates the differences between on-campus and off-campus microbial communities in terms of richness, structure, enrichment, pathogenicity, and the presence of antibiotic-resistant and hemolytic bacteria, utilizing both metagenomic sequencing and bacterial isolation techniques. In brief, our investigation revealed three primary findings: (1) a distinct microbial divergence between on- and off-campus environments; (2) a high-risk reservoir of multi-drug resistant and hemolytic *Staphylococcus* specifically enriched on campus; and (3) a ‘microbial bridge’ effect where social hubs facilitate the exchange of resistance determinants between isolated populations. These results underscore the role of managed human environments as significant but underappreciated reservoirs for AMR.

Despite the relatively small sampling radius, significant differences in microbial communities were observed. These disparities can largely be attributed to the distinct human activity groups within the two environments, which arose from the entry control policies implemented following the COVID-19 pandemic. These policies restricted non-campus individuals from entering the university, resulting in a clear distinction between the campus and surrounding area populations. Such real-time isolation has led to distinct microbial profiles, influenced by the unique characteristics of the human groups present in each environment. Specifically, the on-campus population represents a relatively homogeneous group of healthy young adults with synchronized habits, whereas the off-campus community encompasses a more heterogeneous demographic with a broader spectrum of health statuses, likely contributing to the greater diversity of opportunistic pathogens observed in that setting. β-diversity analysis revealed that ecological distances among locations were related to the frequency of campus personnel visiting these sites. In Figure 3, subway stations, shopping centers, and restaurants-frequent destinations for campus members-exhibited ecological distances close to those of on-campus sites. The shopping center, being the closest in ecological distance to the off-campus, may be explained by the fact that many of its employees live off-campus and can freely enter the site. This pattern is further elucidated by the “human-mediated microbial bridge” created by campus members visiting off-campus hubs for social and essential activities. High-contact surfaces in these centers facilitate a reciprocal exchange of skin-associated and environmental microbiota, effectively homogenizing the microbial profiles between the two environments despite administrative isolation. The microbial enrichment patterns are also noteworthy. Several opportunistic pathogens, particularly within the genus *Corynebacterium* were identified off-campus, including *Corynebacterium ureicelerivorans* and *Corynebacterium glucuronolyticum* [30,31]. Both species have been linked to urinary tract infections, which is particularly concerning given that samples were primarily collected from toilet-hand contact surfaces, suggesting a potential pathway of hand-mediated transmission. This suggests a potential pathway of hand-mediated transmission. The individual pathways of such transmission are likely facilitated by the unrestricted movement of diverse populations in off-campus areas. Unlike the controlled campus environment, public off-campus facilities experience higher turnover and more varied human-to-surface interactions. Such behavioral dynamics, combined with potentially less standardized hygiene practices in these public spaces, may allow high-contact interfaces, such as toilet handles, to act as critical transmission nodes, promoting the sequential transfer of opportunistic pathogens between environment and humans [32].

A significant proportion (84.7%) of the bacterial strains isolated in this study were resistant to at least one antibiotic, emphasizing the widespread presence of AMR on high-contact surfaces. Compared to previous research, the resistance prevalence in our isolates was substantially higher than that reported in food-production settings like slaughterhouses [33], yet more moderate than the levels found in concentrated reservoirs such as wastewater [34] (Table 1).

These discrepancies likely reflect the distinct selective pressures inherent to each environment. Whereas wastewater systems act as a significant sink for diverse antimicrobial residues and genetic determinants [4], daily hand-contact surfaces are characterized by more intermittent chemical exposure but significantly higher human-mediated microbial exchange. In line with previous research, we observed that the detection rates of resistant and hemolytic bacteria were higher on-campus than off-campus. We speculate that the relatively homogeneous and smaller on-campus population may promote microbial evolution and exchange, coupled with the taxonomic clustering of genera such as *Corynebacterium* and *Staphylococcus* on shared surfaces, creates a hotspot for microbial adaptation. Such high congeneric density effectively lowers ecological barriers for the horizontal gene transfer of resistance and virulence determinants, thereby driving the development of the more pronounced resistance and hemolytic phenotypes observed in our campus isolates. The presence of hemolytic bacteria is particularly concerning, as it signals the acquisition of virulence traits that could contribute to the pathogenicity of these organisms. A recent study [35] reported that antibiotic resistance could disrupt the balance of gut microbiota and pose health risks, reinforcing the urgent need for better environmental surveillance.

Among the Gram-positive isolates, *Staphylococcus* species predominated and exhibited an alarmingly high resistance rate of 93.2% on campus. Notably, high resistance was observed against clinically essential antibiotics, including rifampicin (24.5%) and erythromycin (64.1%), which are cornerstone treatments for tuberculosis and respiratory infections, respectively. The erythromycin resistance levels on campus closely mirror those reported in wastewater (67.92%), suggesting a persistent selection pressure driven by human healthcare practices. Conversely, the lower rifampicin resistance compared to slaughterhouse settings reflects the distinct evolutionary trajectories of AMR in human communities versus food-production environments, where veterinary-specific antibiotic use prevails. The enrichment of *Staphylococcus* in combination with high abundances of MRS-associated resistance genes at multiple campus sites suggests that the campus environment may harbor, or at least facilitate, the emergence of MRS. The concurrent detection of *mecA*, *blaZ*, and *pbp2m* genes, coupled with the high contact frequency of campus surfaces, creates conditions conducive to the spread of MRS within the university community. Whole-genome sequencing of seven *Staphylococcus* isolates revealed that 71.4% were resistant to trimethoprim, despite the absence of known resistance genes. This discrepancy could be explained by the presence of novel resistance mechanisms, such as uncharacterized *dfr* genes [36] or efflux pump systems [37]. One isolate carried a *qac* gene, highlighting how exposure to sublethal disinfectant concentrations, often due to organic matter interference, can co-select for low-level antibiotic tolerance [37]. Strategically, this necessitates mitigating sublethal niches to prevent biocide-driven co-selection and monitoring early-stage growth dynamics to track the slow emergence of resistance before it reaches clinically significant thresholds.

While this study presents a strong case for the distinct microbial divergence between on-campus and off-campus environments, the results are constrained by the relatively small sampling radius. Consequently, the findings may primarily reflect the specific socio-ecological dynamics of the studied location. To enhance the generalizability of these conclusions, future research should incorporate larger sample sizes and greater geographical diversity. Additionally, accounting for seasonal variations would provide more robust evidence and a more comprehensive understanding of microbial community shifts at the university-urban interface.

## 5. Conclusions

This study highlighted significant differences in the microbial communities between on- and off-campus environments within a 1.5 km radius. Off-campus areas exhibited higher microbial richness and greater diversity of pathogenic species, while on-campus environments, though less diverse, were dominated by resistant and hemolytic bacteria, particularly *Staphylococcus* species and MRS-related resistance genes. Significantly, identifying plasmid-mediated high clinical-risk carbapenemase genes highlights a severe public health threat that surpasses typical opportunistic pathogens. The widespread presence of plasmid-borne resistance genes further underscores the potential for extensive transmission of antimicrobial resistance. These findings emphasize the health risks posed by both environments, stressing the urgent need for enhanced disinfection protocols and continuous microbial monitoring in densely populated university campuses to mitigate the spread of pathogenic and resistant microorganisms.

## Figures and Tables

**Table 1:** Comparison of antibiotic resistance prevalence across different study environments.

Resistance Category	On-Campus	Slaughterhouse [33]	Wastewater [34]
Overall Resistance	84.7%	60.1%	-
Cephalosporins	33.3%	-	71.25%
Erythromycin	55.7%	-	67.92%
Chloramphenicol	11.8%	-	24.58%

Overall resistance refers to the percentage of isolates resistant to at least one of the tested antibiotics. Data for comparison were adapted from [33] (Enterobacteriaceae isolates from a slaughterhouse) and [34] (isolates from an Indian wastewater reservoir). ‘-’ indicates that data for the specific antibiotic class were not reported in the cited study.

## Data Availability

The original data presented in the study are openly available in Genbank under accession numbers PRJNA1307202.

## Supplementary Materials

The following supporting information can be downloaded at: https://www.mdpi.com/article/10.3390/microorganisms14030557/s1, Figure S1: β-diversity and ecological distance analyses of microbial communities.; Figure S2: LEfSe analysis of non-pathogenic taxa enrichment patterns across campus environments; Figure S3: Strong hemolytic activity of selected *Bacillus* bacteria; Table S1: Raw metagenomic sequencing data statistics; Table S2: Antibiotic resistance gene analysis matrices; Table S3: Antibiotic drug sensitivity results of culturable pathogenic microorganisms.

## Abbreviations

The following abbreviations are used in this manuscript:

ARGs	Antibiotic resistance genes
MRSA	Methicillin-resistant *Staphylococcus aureus*
MRS	Methicillin-resistant *Staphylococcus*
AMR	Antimicrobial resistance
HGT	Horizontal Gene Transfer
PCoA	Principal Coordinate Analysis
NMDS	Non-metric multidimensional scaling
LEfSe	LDA Effect Size
OC-EDC-N	Off-campus express delivery center—North
OC-EDC-S	Off-campus express delivery center—South
OC-RES	Off-campus restaurants
SUB	Subway station
OC-BC	Off-campus Business Center
GYM	Gymnasium
TB	Teaching Building
MLB	Microbiological Laboratory Building
ELB	Engineering Laboratory Building
ON-RES	On-campus restaurants
ON-EDC	On-campus express delivery center
DORM	Student dormitory
ON-BC	On-campus Business Center
