# Supplementary material for: Divergent Microbial Community and Pathogenicity at a University-Urban Interface: A Comparative Analysis
Source: Microorganisms. 2026 Feb 28;14(3):557. doi: 10.3390/microorganisms14030557 (PMC13029079; doi:10.3390/microorganisms14030557)
Supplement: Supplementary file 1 [file microorganisms-14-00557-s001.zip › Supplementary_Materials_Microorganisms.pdf]

1. Supplementary Figures

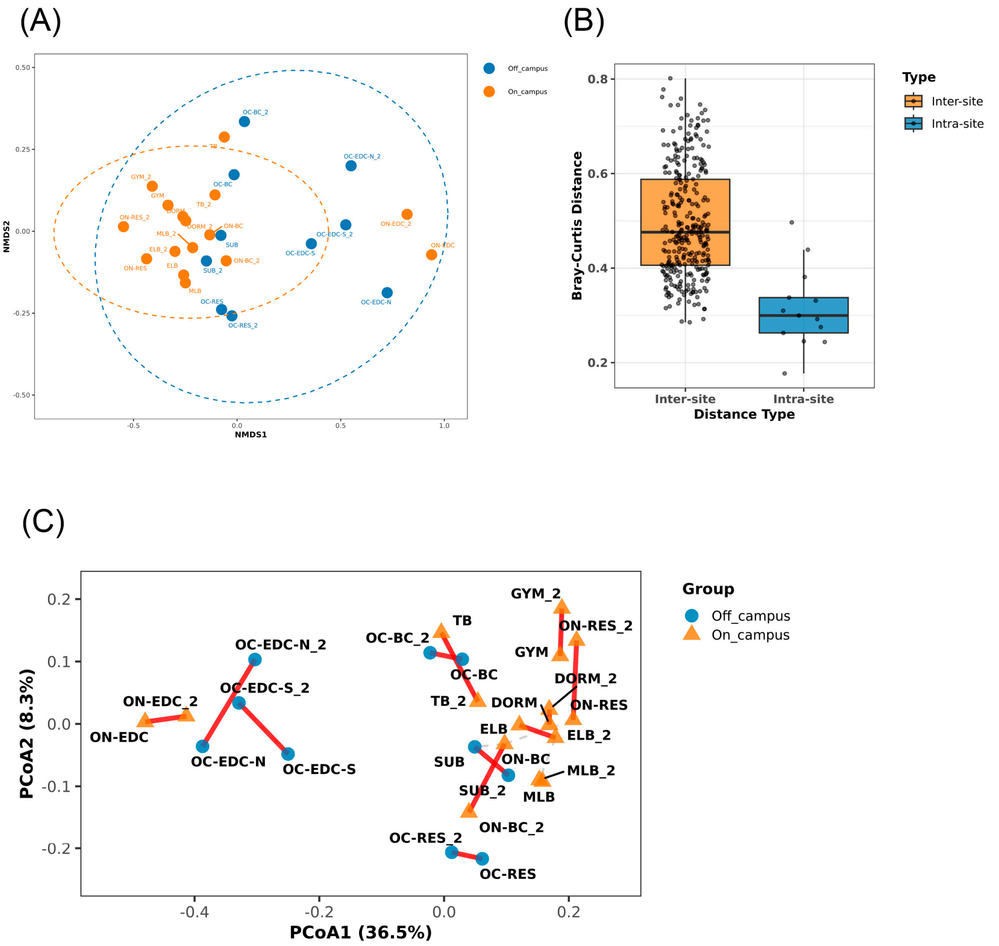

Figure S1. Beta diversity and ecological distance analyses of microbial communities. (A) Non-metric multidimensional scaling (NMDS) based on Bray-Curtis dissimilarity showing significant compositional differences between on- and off-campus environments (PERMANOVA, 999 permutations, P = 0.017). (B) Box plot comparing intra-site versus inter-site Bray-Curtis dissimilarity values. Boxes show median, quartiles, and 1.5× interquartile range; outliers shown as individual points. Permutation test (9,999 permutations) revealed significantly lower intra-site distances ($P < 0.001$, Cohen's d = -1.85), supporting ecological consistency within sampling locations. (C) Principal Coordinate Analysis (PCoA) ordination with distance connections. Blue circles indicate off-campus samples; orange triangles indicate on-campus samples. Red solid lines connect samples from the same sampling location (intra-site); gray dashed lines show nearest inter-site connections (15th percentile of shortest distances).

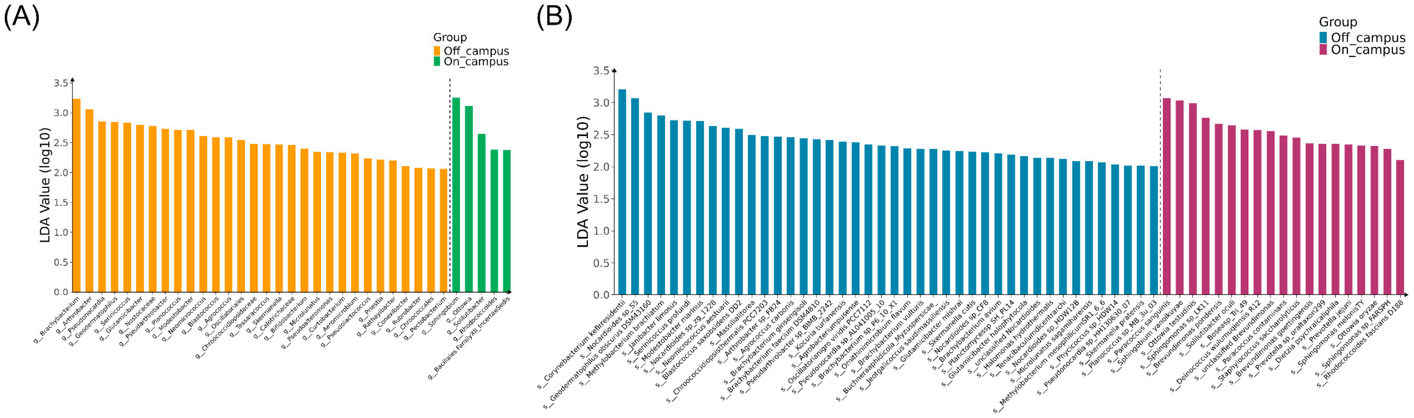

Figure S2. LEfSe analysis of non-pathogenic taxa enrichment patterns across campus environments. (A) Genus-level low-risk taxa. Significantly enriched genera with lower pathogenic potential in on-campus vs off-campus environments. (B) Species-level low-risk taxa. Significantly enriched species with lower pathogenic potential in on-campus vs off-campus environments. All analyses show significant differential enrichment (LDA > 2.0, $P < 0.05$) determined using Kruskal-Wallis test with Wilcoxon rank-sum pairwise comparisons. Taxa highlighted in red represent clinically relevant pathogens discussed in the manuscript. For all panels, the x-axis represents taxonomic information and the y-axis represents LDA scores.

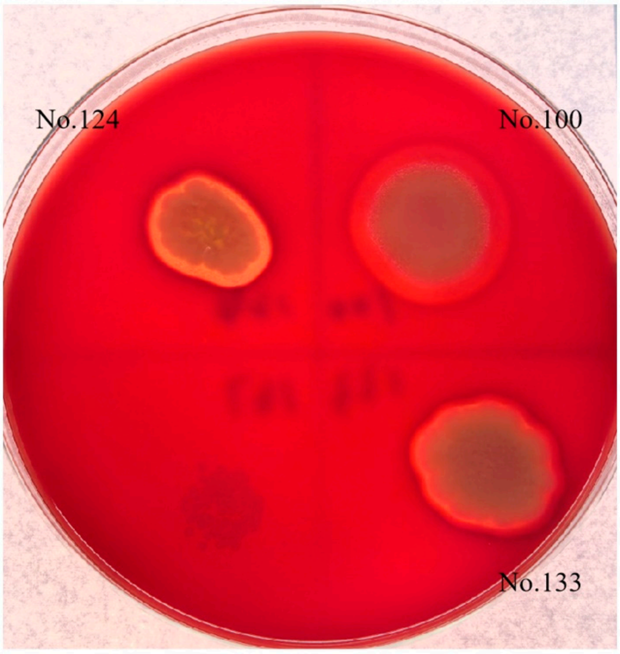

Figure S3. Strong hemolytic activity of selected *Bacillus* bacteria. The figure shows a schematic diagram of hemolytic activity after overnight culture of three Bacillus strains that were diluted and spotted on blood agar plates for overnight incubation. Strain numbers are labeled near the spotted bacterial suspension, where isolated bacterial strains No. 100, 124, and 133 exhibit strong hemolytic activity.

2. Supplementary Tables

Table S1. Raw metagenomic sequencing data statistics. This table lists the library construction names, raw and clean reads/bases (Raw/Clean Reads, Raw/Clean Base), post-filtering efficiency (Effective), average sequencing error rate (Error), base quality proportions (Q20, Q30), and GC content for each sample. All samples were sequenced with approximately 10 Gb of raw data.

Table S2. Antibiotic resistance gene analysis matrices. This Excel file contains four worksheets: Sheet 1: ARG Category Abundance Matrix. Header rows show sample names, sampling times, and site groupings (On-campus/Off-campus). Rows display ARG categories with abundance values (FPKM) and statistical comparisons between groups. Sheet 2: ARG Frequency Matrix. Columns show gene names (with subtypes, e.g., *erm(C)*), resistance classes (multiple classes separated by "/"), and occurrence frequency across samples. Sheet 3: Plasmid-borne ARG Frequency Matrix. Same structure as Sheet 2, limited to plasmid-located ARGs. Sheet 4: Whole Genome Sequencing Assembly and ARG Localization. Taxonomy from Kraken annotation, resistance genes from AMRFinderPlus, gene locations distinguished by PlasFlow.

Table S3. Antibiotic drug sensitivity results of culturable pathogenic microorganisms. This table shows eight different types of antibiotics selected for bacteria of different genera, with hemolytic testing performed on *Staphylococcus* and *Bacillus* bacteria. *Escherichia coli* ATCC 25922 was selected as the standard strain for gram-negative bacteria; *Staphylococcus aureus* ATCC 25923 as the standard strain for *Staphylococcus*; and *Enterococcus faecalis* ATCC 29212 as the standard strain for *Enterococcus* and others. In resistance results, R indicates resistant, I indicates intermediate, and S indicates susceptible. For hemolytic activity testing, "+" indicates hemolytic activity; "++" indicates strong hemolytic activity; no marking indicates no hemolytic activity.
